# *Bothrops* snake venoms and their isolated toxins, an L-amino acid oxidase and a serine protease, modulate human complement system pathways

**DOI:** 10.1186/s40409-015-0026-7

**Published:** 2015-08-13

**Authors:** Lorena Rocha Ayres, Alex dos Reis Récio, Sandra Mara Burin, Juliana Campos Pereira, Andrea Casella Martins, Suely Vilela Sampaio, Fabíola Attié de Castro, Luciana Simon Pereira-Crott

**Affiliations:** Departamento de Análises Clínicas, Toxicológicas e Bromatológicas, Faculdade de Ciências Farmacêuticas de Ribeirão Preto, Universidade de São Paulo (USP), Avenida do Café, s/n, Ribeirão Preto, SP CEP 14040–903, Brasil

**Keywords:** *Bothrops jararacussu*, *Bothrops pirajai*, Chemotaxis, Complement system, Kinetic microassay, L-amino acid oxidase, Serine protease, Snake venom

## Abstract

**Background:**

Activation of the complement system plays an important role in the regulation of immune and inflammatory reactions, and contributes to inflammatory responses triggered by envenomation provoked by *Bothrops* snakes. The present study aimed to assess whether *Bothrops jararacussu* and *Bothrops pirajai* crude venoms and their isolated toxins, namely serine protease (BjussuSP-I) and L-amino acid oxidase (BpirLAAO-I), modulate human complement system pathways.

**Methods:**

Lyophilized venom and toxin samples solubilized in phosphate buffered saline were diluted in appropriate buffers to evaluate their hemolytic activity on the alternative and classical pathways of the complement system. Venom- and toxin-treated normal human serum was added to the erythrocyte suspension, and the kinetic of hemolysis was measured spectrophotometrically at 700 nm. The kinetic 96-well microassay format was used for this purpose. We determined the t^½^ values (time required to lyse 50 % of target erythrocytes), which were employed to calculate the percentage of inhibition of the hemolytic activity promoted by each sample concentration. To confirm complement system activation, complement-dependent human neutrophil migration was examined using the Boyden chamber model.

**Results:**

At the highest concentration tested (120 μg/mL), *B. jararacussu* and *B. pirajai* crude venoms inhibited the hemolytic activity of the classical pathway (65.3 % and 72.4 %, respectively) more strongly than they suppressed the hemolytic activity of the alternative pathway (14.2 and 13.6 %, respectively). BjussuSP-I (20 μg/mL) did not affect the hemolytic activity of the classical pathway, but slightly decreased the hemolytic activity of the alternative pathway (13.4 %). BpirLAAO-I (50 μg/mL) inhibited 24.3 and 12.4 % of the hemolytic activity of the classical and alternative pathways, respectively. Normal human serum treated with *B. jararacussu* and *B. pirajai* crude venoms induced human neutrophil migration at a level similar to that induced by zymosan-activated normal human serum.

**Conclusion:**

Together, the results of the kinetics of hemolysis and the neutrophil chemotaxis assay suggest that pre-activation of the complement system by *B. jararacussu* and *B. pirajai* crude venoms consumes complement components and generates the chemotactic factors C3a and C5a. The kinetic microassay described herein is useful to assess the effect of venoms and toxins on the hemolytic activity of the complement system.

## Background

Snakes of the genus *Bothrops* account for the majority of snakebites in Latin America [1]. Several signs and symptoms follow *Bothrops* envenomation, which are associated with both local effects – such as myonecrosis, hemorrhage, edema and dermonecrosis – and systemic disorders – characterized by coagulopathy, hemorrhage, hypertension, cardiovascular shock and acute renal failure [2].

*Bothrops* snake venoms trigger a typical local inflammatory response that involves edema and the subsequent mobilization of leukocytes. However, it is still not clear how *Bothrops* snake venoms elicit leukocyte recruitment, which is essential to restore tissue homeostasis and repair the injured sites. Studies on *B. asper* venom suggest that the activation of the complement system (CS) is one of the mechanisms underlying this event [3].

*Bothrops* venoms are a complex mixture of components including phospholipases A_2_, metalloproteases, serine proteases (SPs) and L-amino acid oxidases (LAAOs) that exert different pharmacological and biochemical activities [4–6]. There are evidences that the inflammatory response elicited by *Bothrops* toxins is mainly mediated by phospholipases A_2_ and metalloproteases [4, 7]. However, the possible participation of snake venom SPs and LAAOs in the course of the inflammatory response, including activation of the CS, should not be discarded [5].

Human CS is composed of about 35 to 40 proteins and glycoproteins present in blood plasma or on cell surfaces [8]. The CS performs important biological functions in the maintenance and regulation of immune and inflammatory reactions. Its proteins interact with each other in a highly regulated manner to promote inflammation and destroy invading microorganisms and foreign cells. As the CS has the potential to damage host tissues, regulatory proteins tightly control the activation and activity of this system [9].

The complement system can be activated via three distinct pathways – classical (CS-CP), alternative (CS-AP) or lectin (CS-LP) – depending on the stimulus type. The CS-CP is activated when C1, the first component of the CS cascade, binds to an antibody molecule complexed with an antigen. The CS-AP is activated by spontaneous hydrolysis of the C3 component and subsequent deposition of C3 fragments on activating surfaces [10]. The CS-LP, more recently discovered, is activated through recognition of carbohydrates on the surface of microorganisms by mannose binding lectins [11]. The CS activation entails sequential proteolytic reactions, a process called CS cascade, which generates products with a variety of biological activities such as anaphylaxis, chemotaxis, opsonization, solubilization of immune complexes, and modulation of the immune response [12].

Although the effect of snake venoms on the CS activity has already been reported, much remains to be investigated, in particular with regard to the action of SPs and LAAOs [13–15]. Snake venom SPs act mainly on components of the coagulation cascade, which in turn leads to a hemostatic imbalance [16, 17]. Flavoxobin, a SP from *Trimeresurus flavoviridis* snake venom, specifically cleaves the human complement protein C3, thus acting as a C3 convertase enzyme [18]. The biological effects of snake venom LAAOs usually proceed through induction of apoptosis, cytotoxicity, and inhibition or induction of both platelet aggregation and microbicidal activity [19–23].

To date, there are no reports on the action of the L-amino acid oxidase isolated from *B. pirajai* venom (BpirLAAO-I) on the complement system. In order to broaden the existing knowledge on the biological properties of *Bothrops* snake venom, the present study aims to assess whether *B. jararacussu* (Bjussu) crude venom and its serine protease (BjussuSP-I), as well as *B. pirajai* (Bpir) crude venom and its LAAO (BpirLAAO-I), modulate the human CS pathways.

## Methods

### Snake venoms and toxins

The lyophilized Bpir and Bjussu crude venoms were acquired from the snake house of Bioagents Bioactive Proteins Ltd. (Batatais, SP, Brazil). BpirLAAO-I was purified by the three-step chromatographic process reported by Izidoro *et al.* [24], which employed Sephadex G-75, Benzamidine-Sephadex, and Phenyl-Sepharose as stationary phases. The purity of the final preparation was higher than 95 %. The BpirLAAO-I enzymatic activity was determined before performing the experiments. BjussuSP-I was isolated by a three-step chromatographic process that used Sephacryl S-200, Benzamidine Sepharose, and C2/C18 as stationary phases, as described by Menaldo *et al*. [17].

### Animals

Two adult female New Zealand white rabbits, weighing approximately 3.3 kg, and two adult sheep were obtained from the Central Animal Facility of the University of São Paulo, campus of Ribeirão Preto (USP-RP). The rabbits were kept in the vivarium at the School of Pharmaceutical Sciences of Ribeirão Preto (FCFRP/USP).

Blood was collected from the rabbit’s central ear artery or the sheep’s jugular vein into an equal volume of modified Alsever’s solution as anticoagulant, and further employed to assay the hemolytic activity of the CS.

### Normal human serum (NHS)

Ten milliliter of blood from healthy volunteers of both genders, aged from 20 to 50 years, were collected in the absence of anticoagulants to obtain normal human serum (NHS). The sera were separated by centrifugation at 500 × *g* for ten minutes at 4 °C, pooled, aliquoted into polyethylene tubes, and frozen at −80 °C. NHS pool was employed to evaluate the immunomodulatory activity of Bjussu and Bpir crude venoms and their isolated toxins on the CS activity and the CS-dependent neutrophil chemotaxis.

### Human CS modulation by venoms and toxins

#### Preparation of sheep erythrocytes to assay the CS-CP/LP activity

Sheep erythrocyte suspension was prepared as previously described [17]. Briefly, the anticoagulated sheep blood was diluted in triethanolamine (TEA) buffer containing Ca^2+^ and Mg^2+^ (TEA-Ca^2+^-Mg^2+^ buffer) and centrifuged. After discarding the plasma and buffy coat, the erythrocytes were washed and suspended to a concentration of approximately 5 % (1.2 × 10^9^ cells/mL). This suspension was mixed with an appropriate dilution of hemolysin and incubated for 15 min, at 4 °C. Final absorbance of the suspension was adjusted to an optical density ranging from 0.7 to 0.8 at 700 nm (SpectraMax® Plus spectrophotometer, Molecular Devices, USA).

#### Preparation of rabbit erythrocytes to assay the CS-AP activity

Rabbit erythrocyte suspension was prepared as described [17]. Briefly, the anticoagulated rabbit blood was filtered, diluted in an equal volume of a Ca^2+^ and Mg^2+^-chelating solution composed of TEA and EDTA (ethylenediamine tetraacetic acid), and incubated for 15 min, at 37 °C. After washing three times with TEA-Mg^2+^ buffer, the erythrocytes were suspended in modified Alsever’s solution supplemented with 0.05 % sodium azide, in a volume equal to twice the original blood volume. Finally, the erythrocyte suspension was divided into aliquots and stored at 4 °C.

Prior to use in the hemolytic assay, the erythrocytes were washed three times with TEA-EGTA-Mg^2+^ buffer [EGTA: ethylene glycol-bis (2-aminoethylether)-N,N,N',N'-tetraacetic acid], and suspended in this buffer. Final absorbance of the suspension was adjusted to an optical density ranging from 0.7 to 0.8 by spectrophotometric reading at 700 nm.

#### Measurement of the hemolytic activity of the CS using the kinetic method

The residual hemolytic activity of the CS in NHS treated with either crude venoms or isolated toxins was assessed through the kinetic 96-well microassay, as previously described [17]. Bjussu and Bpir crude venoms (3.1-120 μg/mL), and the toxins BjussuSP-I (0.6-20 μg/mL) and BpirLAAO-I (1.6-50 μg/mL) were diluted in appropriate buffers – TEA-Ca^2+^-Mg^2+^ buffer to assay the CS-CP/LP activity or TEA-EGTA-Mg^2+^ buffer to assay the CS-AP activity – to the concentrations indicated in parentheses. These samples were incubated with NHS in 96-well microplates for one hour, at 37 °C, in a final volume of 200 μL. Aliquots of sensitized sheep erythrocytes (CS-CP/LP) or rabbit erythrocytes (CS-AP) were added to the wells and the kinetics of hemolysis was followed by uninterrupted recording of absorbance at 700 nm for 15 min (SpectraMax Plus Microplate Reader, Molecular Devices, USA).

The time-course curve of hemolysis was used to determine the time required to lyse 50 % of the erythrocytes (t^½^), which corresponds to the time required for the absorbance to decline to half of its initial value. The t^½^ value is directly proportional to the percentage of suppression of the hemolytic activity of the CS, which was calculated for each sample concentration, as follows [17, 25]:

$$ Hemolysis\ inhibition\left(\%\right)=100\hbox{-} \frac{t^{\frac{1}{2}}\kern0.10em  control\  wells\times 100}{t^{\frac{1}{2}}\kern0.15em  sample\  wells} $$

These percentages of inhibition values were fitted to a linear regression curve to determine the IC_50_ value, which is the venom or toxin concentration that inhibits 50 % of hemolysis. To obtain accurate IC_50_ values, at least one of the values of percentage of inhibition must be greater than 50 %.

### Assay of CS-dependent neutrophil chemotaxis

#### Serum treatment

Aliquots of NHS were treated with zymosan or venom as previously described [26]. The amount of crude venom used in the chemotaxis assay was calculated based on the amount of venom that best inhibited the hemolytic activity of CS-CP/LP, 120 μg/mL, which corresponds to 3.3 μg of venom for each 1 μL of NHS. Briefly, Bjussu and Bpir crude venoms (400 μg) diluted in TEA-Ca^2+^-Mg^2+^ buffer (final volume of 100 μL) were incubated for 40 min, at 37 °C, with 120 μL of: NHS, heat-inactivated NHS (56 °C, 30 min), or TEA-Ca^2+^-Mg^2+^ buffer. Zymosan-activated NHS and NHS incubated with TEA-Ca^2+^-Mg^2+^ buffer were used as positive and negative controls, respectively. To inactivate residual complement, the supernatants were collected and heated to 56 °C for 30 min. Finally, the supernatants were diluted 1:5 in Hanks buffered saline solution (HBSS) to be used in the chemotaxis assay.

#### Human neutrophils isolation

Human neutrophils were isolated from peripheral blood of healthy volunteers using the Ficoll-Hypaque discontinuous density gradient method. Histopaque-1077 was layered over Histopaque-1119, and the blood samples were processed according to the manufacturer's instructions (Sigma Diagnostics, Inc., USA). After washing the cell pellets with HBSS, the concentration of the neutrophil suspension was adjusted to 2 × 10^6^ cells/mL.

#### Chemotaxis assay

The neutrophil chemotaxis assay was performed using a modified Boyden chamber, as previously described [27]. Briefly, the lower chamber was filled with 200 μL of treated NHS and covered with a filter (diameter: 13 mm, pore size: 3 μm, SSWPO1300, Millipore Corp., USA). The upper compartment was filled with 300 μL of neutrophil suspension. After 30 min of incubation at 37 °C in humidified air, the filters were removed, fixed in 2-propanol, stained with Harris hematoxylin, dehydrated in 2-propanol, cleared with xylene, and mounted with Entellan® mounting media (Merck, Germany). The neutrophil migration was determined by the leading front technique, which measures the greatest distance in micrometers crossed by three cells per field with a 100× magnification [28]. At least ten fields per filter were examined.

### Ethics committee approval

The Research Ethics Committee of FCFRP/USP approved the experimental procedures involving human cells and sera, which were registered under CEP/FCFRP n. 125/2008. The Ethics Committee on Laboratory Animal Care and Use at USP-RP approved the animal housing and handling procedures, as well as the experimental protocols involving animal cells, which were registered under CEUA n. 08.1.362.53.0/2008.

### Statistical analyses

Experimental data were analyzed by One-way ANOVA followed by the Bonferroni's post-hoc test, with the aid of the GraphPad Prism Software (version 5.0, GraphPad Software, USA). Values of *p* < 0.05 were considered significant.

## Results

### Modulation of the hemolytic activity of the CS

To assess whether Bjussu and Bpir crude venoms and the isolated toxins BjussuSP-I and BpirLAAO-I modulate the hemolytic activity of the CS, we measured the residual hemolytic activity of CS in sera treated with these samples, using the kinetic microassay. To conduct separate analysis of the sample effects on CS-CP/LP and CS-AP, we employed sheep erythrocytes suspended in TEA-Ca^2+^-Mg^2+^ buffer and rabbit erythrocytes suspended in TEA-EGTA-Mg^2+^ buffer, respectively. The obtained results are reported below, where the increase in t^½^ values means suppression of the hemolytic activity of CS.

#### Bjussu and Bpir crude venoms selectively suppress the hemolytic activity of the CS

**Fig. 1:**
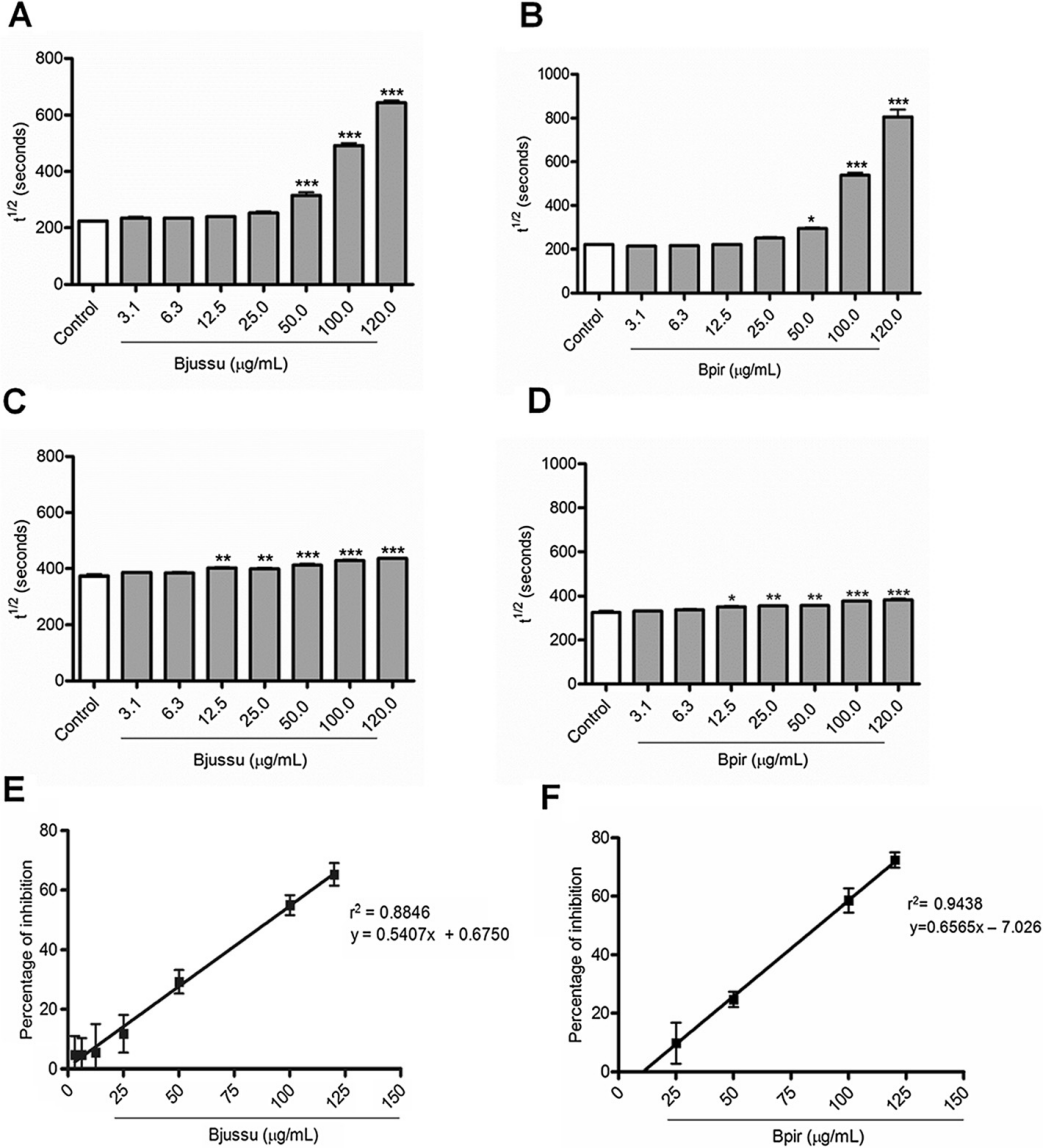
Effect of Bjussu and Bpir crude venom on the hemolytic activity of the complement system. This figure depicts the concentration-dependent inhibitory effect of (**a**, **c**, and **e**) Bjussu and (**b**, **d**, and **f**) Bpir crude venoms on the hemolytic activity of the (**a**, **b**, **e**, and **f**) classical and (**c** and **d**) alternative pathways of the complement system. Panels **a** to **d**: Control represents normal human serum incubated with buffer alone. Data are expressed as the mean ± standard deviation of the t^½^ values obtained for each venom concentration, based on three (CS-CP/LP) or two (CS-AP) independent experiments assayed in triplicate. *p < 0.05, **p < 0.001, or ***p < 0.0001 vs. control. Panels **e** and **f**: Linear regression graph, where the X-values represent the amount of (**e**) Bjussu and (**f**) Bpir crude venom (in μg/mL) and the Y-values represent the mean percentages of hemolytic activity inhibition. The IC_50_ values were calculated from three independent experiments. Bjussu: *Bothrops jararacussu*; Bpir: *Bothrops pirajai*; t^½^: time required to lyse 50 % of erythrocytes

Bjussu and Bpir crude venoms augmented the time required to lyse 50 % of erythrocytes (t^½^) values (i.e. diminished the hemolytic activity) for both CS pathways studied, in a concentration-dependent manner. Such increase was statistically significant at concentrations higher than 50 μg/mL and 12.5 μg/mL for the CS-CP/LP and the CS-AP, respectively (Fig. 1a-d). Bjussu and Bpir crude venoms similarly inhibited the hemolytic activity of the CS-CP/LP, yielding mean IC_50_ values of 91.2 μg/mL and 86.9 μg/mL, respectively (Fig. 1e-f).

It was not possible to determine the IC_50_ values for the CS-AP because the highest Bjussu and Bpir crude venom concentration tested (120 μg/mL) suppressed less than 50 % of the hemolytic activity: 14.2 and 13.6 %, respectively. At this concentration, Bjussu and Bpir crude venom reduced the hemolytic activity of the CS-CP/LP by 65.3 and 72.4 %, respectively. Therefore, Bjussu and Bpir crude venoms inhibited the hemolytic activity of the CS-CP/LP more effectively than they inhibited the hemolytic activity of the CS-AP.

#### The isolated toxins BjussuSP-I and BpirLAAO-I inhibit the hemolytic activity of the CS

**Fig. 2:**
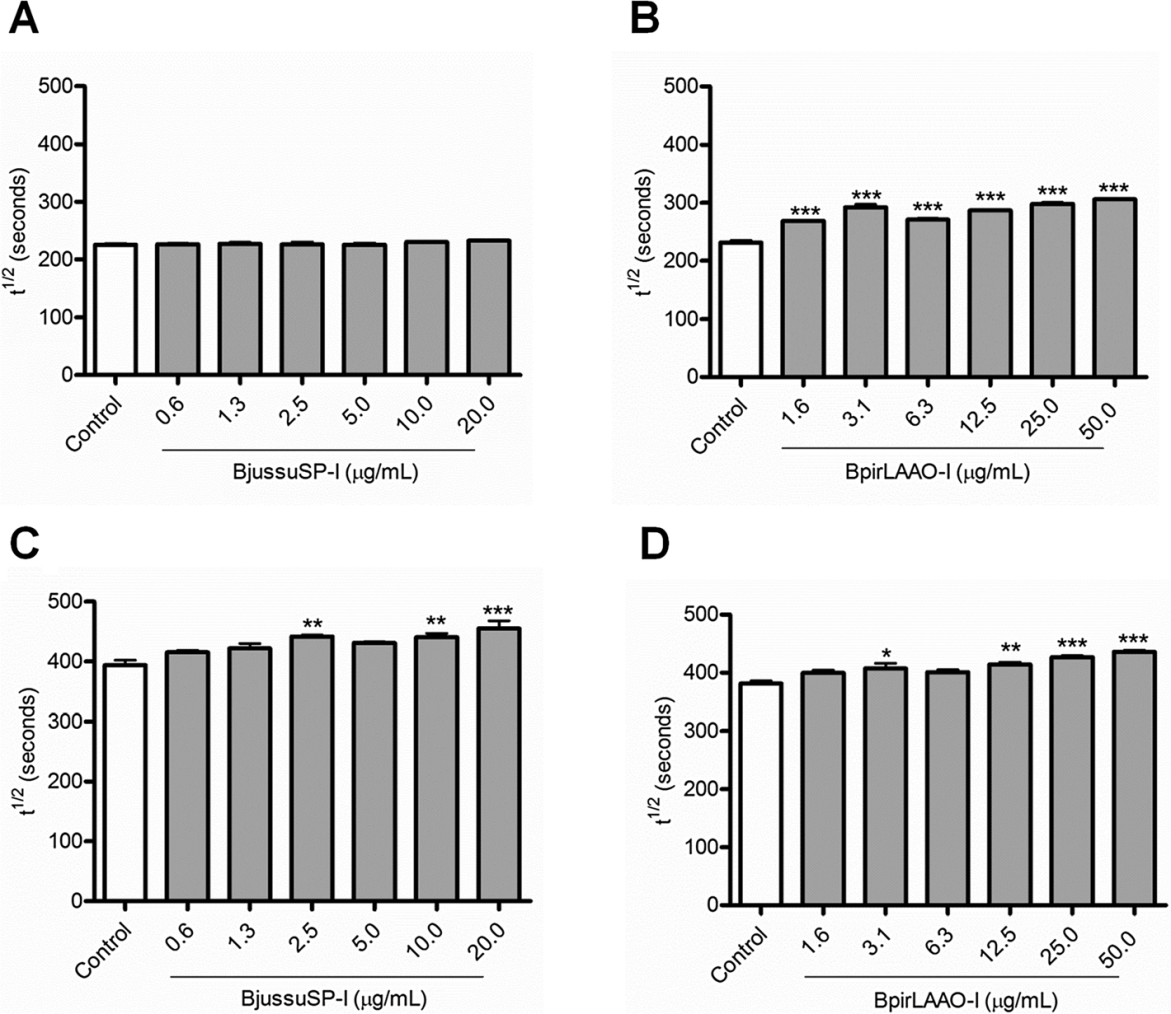
Effect of the toxins BjussuSP-I and BpirLAAO-I on the hemolytic activity of the complement system. This figure depicts the concentration-dependent inhibitory effect of (**a** and **c**) BjussuSP-I and (**b** and **d**) BpirLAAO-I on the hemolytic activity of the (**a** and **b**) classical and (**c** and **d**) alternative pathways of the complement system. Control represents normal human serum incubated with buffer alone. Data are expressed as the mean ± standard deviation of the t^½^ values obtained for each toxin concentration, based on three (CS-CP/LP) or two (CS-AP) independent experiments assayed in triplicate. *p < 0.05, **p < 0.001, or ***p < 0.0001 vs. control. BjussuSP-I: serine protease isolated from *Bothrops jararacussu* crude venom; BpirLAAO-I: L-amino acid oxidase isolated from *Bothrops pirajai* crude venom; t^½^: time required to lyse 50 % of erythrocytes

In the range of concentrations tested (0.6-20 μg/mL), BjussuSP-I did not significantly alter the t^½^ values for the CS-CP/LP (Fig. 2a). On the other hand, this toxin significantly augmented the t^½^ values for the CS-AP at 2.5, 10, and 20 μg/mL (Fig. 2c). BpirLAAO-I significantly increased the t^½^ values for the CS-CP/LP at all concentrations tested (Fig. 2b), and the t^½^ values for the CS-AP at the concentrations of 3.1, 12.5, 25, and 50 μg/mL (Fig. 2d).

Although BjussuSP-I and BpirLAAO-I exerted concentration-dependent effects, it was not possible to determine their IC_50_ values. At the highest concentration tested, BjussuSP-I inhibited the hemolytic activity of the CS-AP by 13.4 %, while BpirLAAO-I suppressed the hemolytic activity of CS-CP/LP and CS-AP by 24.3 and 12.4 %, respectively. Therefore, compared with the crude venoms, the isolated toxins weakly inhibit the hemolytic activity of both pathways of the CS.

### Bjussu and Bpir crude venom-treated sera induce neutrophil chemotaxis

**Fig. 3:**
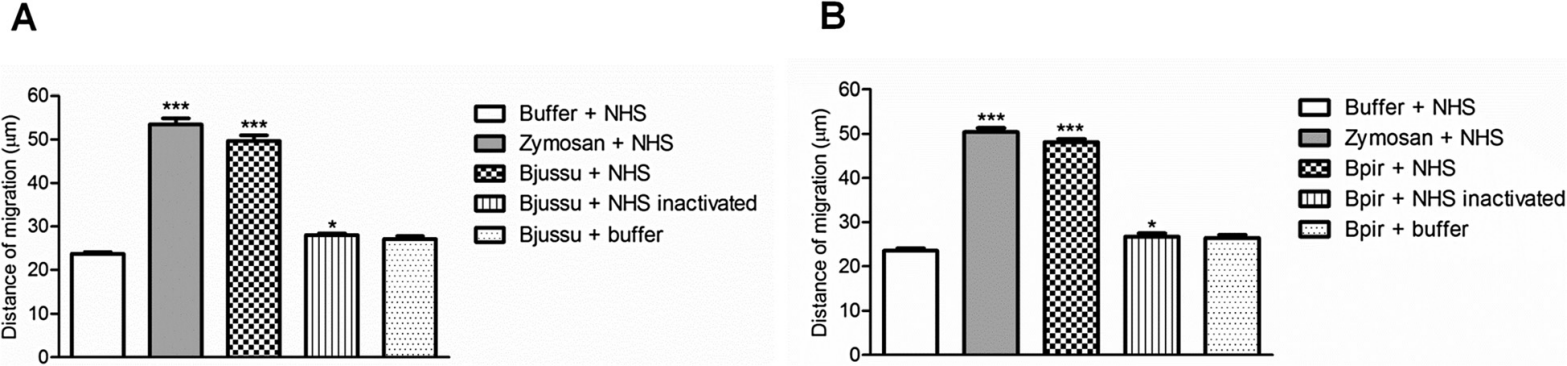
Chemotactic response of neutrophils to serum treated with (**a**) Bjussu and (**b**) Bpir crude venom. Normal human serum (NHS) was treated with crude venom, zymosan (positive control), or TEA-Ca^2+^-Mg^2+^ buffer (negative control). Crude venoms were also incubated with TEA-Ca^2+^-Mg^2+^buffer alone or heat-inactivated NHS. Data are expressed as the mean ± standard deviation of three independent experiments assayed in duplicate. *p < 0.05 or ***p < 0.0001 vs. negative control. Bjussu: *Bothrops jararacussu*; Bpir: *Bothrops pirajai*

Bjussu and Bpir crude venom, the samples that most strongly inhibited the hemolytic activity of CS, were assessed for their ability to modulate the human neutrophil chemotaxis. The venoms alone, i.e. incubated with the CS-CP buffer in the absence of NHS, did not elicit neutrophil migration. The mean distances of migration induced by NHS alone (negative control) were 23.7 μm and 23.5 μm (Fig. 3a and b, respectively).

The Bjussu and Bpir crude venom-treated NHS and zymosan-treated NHS equally induced neutrophil migration (Fig. 3). The mean distances of migration induced by Bjussu and Bpir crude venom-treated NHS were 49.7 μm and 48.1 μm, respectively, and 53.5 μm (Fig. 3a) and 50.4 μm (Fig. 3b) for zymosan-treated NHS. Heat-inactivated NHS treated with Bjussu and Bpir crude venoms slightly stimulated neutrophil migration (28.0 μm and 26.7 μm, respectively). Thus, pre-treatment of NHS with Bjussu and Bpir crude venoms generate chemotactic factors.

## Discussion

Snake venoms consist of complex mixtures of proteins that affect different systems in the human organism, including the CS [1, 14, 15, 29]. Venoms of snakes belonging to the Crotalidae and Viperidae families contain a variety of proteins that modulate the CS activity [30]. These molecules may directly cleave CS proteins such as C2, C3, and C4 and stabilize the C3-convertase of the CS-AP, which in turn amplifies the activation cascade [13, 31, 32]. Consequently, the production of the anaphylatoxins C3a and C5a and other fragments of the CS activation, such as C3b and C4b, is increased [13, 31, 32]. Venoms of snakes from the Elapidae family also contain molecules capable of activating the CS. These venoms reduce the hemolytic activity of the CS and convert C3 into products whose electrophoretic mobilities are distinct from those of the native C3 molecule in human serum [33].

In this study, treatment of NHS with Bjussu and Bpir crude venoms markedly reduced the hemolytic activity of the CS-CP/LP, which indicates that these venoms are potential modulators of this CS pathway. These findings are consistent with another study showing that the hemolytic activity of serum samples incubated for one hour at 37 °C with *B. atrox* venom fractions was reduced by more than 85 % [34]. In contrast, Bjussu and Bpir crude venoms only weakly inhibited the hemolytic activity of the CS-AP.

Regarding the isolated toxin BpirLAAO-I, this is the first study to demonstrate its negative modulatory effect on the hemolytic activity of the CS-CP/LP and CS-AP. This toxin increased the t^½^ values for both CS pathways at almost all the concentrations tested. On the other hand, BjussuSP-I selectively inhibited the hemolytic activity of the CS-AP, at least under the assessed conditions.

A recent study has demonstrated that two Bpir SPs, named BpirSP41 and BpirSP27, similarly inhibit the hemolytic activity of the CS-AP, but modulate the CS-CP/LP at different levels: BpirSP41 suppress the hemolytic activity of this CS pathway more strongly than BpirSP27 [17]. Flavoxobin, a SP isolated from *Trimeresurus flavoviridis*snake venom, activates the human CS-AP and leads to the formation of membrane attack complex and to the release of C3a and C5a. This SP was identified as a heterologous C3 convertase that cleaves C3 directly and selectively to form C3b and C3a [18].

The aforementioned results of the present study were not able to elucidate whether the effects of Bjussu and Bpir crude venoms and the isolated toxins BjussuSP-I and BpirLAAO-I were due to: (i) a prior activation of the CS during pre-incubation of the venom with NHS; (ii) inactivation of CS components; (iii) interference in the CS activation processes; and/or (iv) interaction with CS regulators. Thus, other research strategies are required to unravel the mechanism of action of these *Bothrops* venoms and toxins on the CS.

One strategy relies on the direct or indirect measurement of possible fragments generated by the CS activation. Induction of neutrophil chemotaxis by activated serum is an indirect method to assess the formation of CS products C3a and C5a, which are potent chemoattractants [35]. In this study, NHS treated with Bjussu and Bpir crude venoms elicited neutrophil migration to levels similar to those elicited by zymosan-treated NHS. Compared with the NHS alone, crude venoms alone did not trigger neutrophil migration. These results led us to conclude that crude venoms induced the cleavage of C3 and C5 components of the CS in NHS, generating the chemotactic fragments C3a and C5a. Previous studies have reported that serum treated with *B. asper* and *Tityus serrulatus* venoms were able to induce rat neutrophil migration. The authors have also proposed that the mechanism of action of venoms involved the generation of CS fragments with chemotactic activity [3, 25].

The production of the anaphylatoxins C3a and C5a probably plays a role in *in vivo* inflammatory processes that occur after *Bothrops* envenomation, such as edema, coagulopathies, leukocyte mobilization, generation of reactive oxygen species, and secretion of pro-inflammatory cytokines like tumor necrosis factor alpha, interleukin-6, and interleukin-1 [36].

Together, the results of the hemolytic activity of the CS and the CS-dependent neutrophil chemotaxis demonstrate that Bjussu and Bpir crude venoms activate the CS. Our hypothesis is that the hemolytic activity decrease is due to CS pre-activation during NHS incubation with the venom. At that moment, components of the CS are consumed – and thus generate CS fragments, including anaphylatoxins C3a and C5a – which, in turn, diminishes the amount of CS components available to induce hemolysis. In summary, the CS pre-activation by venom reduces the residual hemolytic activity of NHS.

Furthermore, the kinetic microassay employed in this study has proved to be useful for assessing the modulatory effect of snake venoms and toxins on the hemolytic activity of different CS pathways. Compared with the traditional hemolytic assay, the microassay requires smaller volumes of reagents, and shorten the assay length due to simultaneous analysis of many samples. The results reported herein contribute to a better understanding of the mechanism of action of *Bothrops* venoms and toxins.

## Conclusion

Bjussu and Bpir crude venoms activate the CS-CP/LP and generate the chemotactic factors C3a and C5a. As the isolated toxins BjussuSP-I and BpirLAAO-I weakly inhibited the human CS hemolytic activity, it is still necessary to perform further studies to isolate and identify the Bjussu and Bpir venom components responsible for the observed effects as well as to clarify the mechanisms responsible for their biological effects.

### Ethics committee approval

The Research Ethics Committee of FCFRP/USP approved the experimental procedures involving human cells and sera, which were registered under CEP/FCFRP n. 125/2008. The Ethics Committee on Laboratory Animal Care and Use at USP-RP approved the animal housing and handling procedures, as well as the experimental protocols involving animal cells, which were registered under CEUA n. 08.1.362.53.0/2008.

## Abbreviations

Bjussu: *Bothrops jararacussu*
BjussuSP-I: Serine protease from *Bothrops jararacussu*
Bpir: *Bothrops pirajai*
BpirLAAO-I: L-amino acid oxidase from *Bothrops pirajai*
CS: Complement system
CS-AP: Alternative pathway of the complement system
CS-CP: Classical pathway of the complement system
CS-LP: Lectin pathway of the complement system
EDTA: Ethylenediamine tetraacetic acid
EGTA: Ethylene glycol-bis(2-aminoethylether)-N,N,N',N'-tetraacetic acid
HBSS: Hanks buffered saline solution
IC_50_: sample concentration that inhibits 50 % of hemolysis
LAAO: L-amino acid oxidase
NHS: Normal human serum
SP: Serine protease
TEA: Triethanolamine
t^½^: Time required to lyse 50 % of erythrocytes

## References

[1] Segura A, Castillo MC, Núñez V, Yarlequé A, Gonçalves LR, Villalta M (2010). Preclinical assessment of the neutralizing capacity of antivenoms produced in six Latin American countries against medically-relevant *Bothrops* snake venoms. Toxicon.

[2] Nascimento JM, Franchi GC, Nowill AE, Collares-Buzato CB, Hyslop S (2007). Cytoeskeletal rearrangement and cell death induced by *Bothrops alternatus* snake venom in cultured Madin-Darby canine kidney cells. Biochem Cell Biol.

[3] Farsky SH, Gonçalves LR, Gutiérrez JM, Correa AP, Rucavado A, Gasque P (2000). *Bothrops asper* snake venom and its metalloproteinase BaP-1 activate the complement system. Role in leucocyte recruitment. Mediators Inflamm.

[4] Teixeira CF, Landucci EC, Antunes E, Chacur M, Cury Y (2003). Inflammatory effects of snake venom myotoxic phospholipases A_2_. Toxicon.

[5] Teixeira C, Cury Y, Moreira V, Picolo G, Chaves F (2009). Inflammation induced by *Bothrops asper* venom. Toxicon.

[6] Correa-Netto C, Teixeira-Araujo R, Aguiar AS, Melgarejo AR, De-Simone SG, Soares MR (2010). Immunome and venome of *Bothrops jararacussu*: a proteomic approach to study the molecular immunology of snake toxins. Toxicon.

[7] Teixeira CF, Fernandes CM, Zuliani JP, Zamuner SF (2005). Inflammatory effects of snake venom metalloproteinases. Mem Inst Oswaldo Cruz.

[8] Carroll MV, Sim RB (2011). Complement in health and disease. Adv Drug Deliv Rev.

[9] Wagner E, Frank MM (2010). Therapeutic potential of complement modulation. Nat Rev Drug Discov.

[10] Carroll MC (2004). The complement system in regulation of adaptive immunity. Nat Immunol.

[11] Turner MW (1996). Mannose-binding lectin: the pluripotent molecule of the innate immune system. Immunol Today.

[12] Walport MJ (2001). Complement. First of two parts. N Engl J Med.

[13] Eggertsen G, Fohlman J, Sjöquist J (1980). *In vitro* studies on complement inactivation by snake venoms. Toxicon.

[14] Pidde-Queiroz G, Furtado MF, Filgueiras CF, Pessoa LA, Spadafora-Ferreira M, van den Berg CW (2010). Human complement activation and anaphylatoxins generation induced by snake venom toxins from *Bothrops* genus. Mol Immunol.

[15] Tanaka GD, Pidde-Queiroz G, Furtado MFD, van den Berg C, Tambourgi DV (2012). *Micrurus* snake venoms activate human complement system and generate anaphylatoxins. BMC Immunol.

[16] Serrano SM, Maroun RC (2005). Snake venom serine proteinases: sequence homology vs. substrate specificity, a paradox to be solved. Toxicon.

[17] Menaldo DL, Bernardes CP, Pereira JC, Silveira DS, Mamede CCN, Stanziola L (2013). Effects of two serine proteases from *Bothrops pirajai* snake venom on the complement system and the inflammatory response. Int Immunopharmacol.

[18] Yamamoto C, Tsuru D, Oda-Ueda N, Ohno M, Hattori S, Kim ST (2002). Flavoxobin, a serine protease from *Trimeresurus flavoviridis* (habu snake) venom, independently cleaves Arg726-Ser727 of human C3 and acts as a novel, heterologous C3 convertase. Immunology.

[19] Du XY, Clemetson KJ (2002). Snake venom L-amino acid oxidases. Toxicon.

[20] Samel M, Vija H, Ronnholm G, Siigur J, Kalkkinen N, Siigur E (2006). Isolation and characterization of an apoptotic and platelet aggregation inhibiting L-amino acid oxidase from *Vipera berus berus* (common viper) venom. Biochim Biophys Acta.

[21] Guo C, Liu S, Yao Y, Zhang Q, Sun MZ (2012). Past decade study of snake venom L-amino acid oxidase. Toxicon.

[22] Burin SM, Ayres LR, Neves RP, Ambrósio L, de Morais FR, Dias-Baruffi M (2013). L-amino acid oxidase isolated from *Bothrops pirajai* induces apoptosis in BCR-ABL-positive cells and potentiates imatinib mesylate effect. Basic Clin Pharmacol Toxicol.

[23] Costa TR, Burin SM, Menaldo DL, de Castro FA, Sampaio SV (2014). Snake venom L-amino acid oxidases: an overview on their antitumor effects. J Venom Anim Toxins incl Trop Dis.

[24] Izidoro LF, Ribeiro MC, Souza GR, Sant’Ana CD, Hamaguchi A, Homsi-Brandeburgo MI (2006). Biochemical and functional characterization of an L-amino acid oxidase isolated from *Bothrops pirajai* snake venom. Bioorg Med Chem.

[25] Bertazzi DT, de Assis-Pandochi AI, Talhaferro VL, Caleiro Seixas Azzolini AE, Pereira-Crott LS, Arantes EC (2005). Activation of the complement system and leukocyte recruitment by *Tityus serrulatus* scorpion venom. Int Immunopharmacol.

[26] Ayres LR (2010). Modulação de eventos da imunidade humoral e celular por venenos brutos e componentes dos venenos de *Bothrops jararacussu* e *Bothrops pirajai*. MSc Thesis.

[27] Boyden S (1962). The chemotactic effect of mixtures of antibody and antigen on polymorphonuclear leucocytes. J Exp Med.

[28] Zigmond SH, Hirsch JG (1973). Leukocyte locomotion and chemotaxis. New methods for evaluation, and demonstration of a cell-derived chemotactic factor. J Exp Med.

[29] Barros LC, Soares AM, Costa FL, Rodrigues VM, Fuly AL, Giglio JR (2011). Biochemical and biological evaluation of gyroxin isolated from *Crotalus durissus terrificus* venom. J Venom Anim Toxins incl Trop Dis.

[30] Shoibonov BB, Osipov AV, Kryukova EV, Zinchenko AA, Lakhtin VM, Tsetlin VI (2005). Oxiagin from the *Naja oxiana* cobra venom is the first reprolysin inhibiting the classical pathway of complement. Mol Immunol.

[31] Müller-Eberhard HJ, Fjellström KE (1971). Isolation of the anticomplementary protein from cobra venom and its mode of action on C3. J Immunol.

[32] Gasmi A, Louzir H, Karoui H, el Ayeb M, Dellagi K (1994). Purification from *Vipera lebetina* (desert adder) venom of a protein that depletes human complement. Nat Toxins.

[33] Tambourgi DV, dos Santos MC, Furtado MF, de Freitas MC, da Silva WD, Kipnis TL (1994). Pro-inflammatory activities in elapid snake venoms. Br J Pharmacol.

[34] Rodrigues FG, Petretski JH, Kanashiro MM, Lemos L, Silva WD, Kipnis TL (2004). The complement system is involved in acute inflammation but not in the hemorrhage produced by a *Bothrops atrox* snake venom low molecular mass proteinase. Mol Immunol.

[35] Haas PJ, van Strijp J (2007). Anaphylatoxins: their role in bacterial infection and inflammation. Immunol Res.

[36] Luna KPO, da Silva MB, Pereira VRA (2011). Clinical and immunological aspects of envenomation by *Bothrops* snakes. J Venom Anim Toxins incl Trop Dis.

